# Prostate Carcinoma and Pleural Mesothelioma: An Extremely Rare Co-occurrence

**DOI:** 10.7759/cureus.4655

**Published:** 2019-05-14

**Authors:** Tej P Soni, Sweta Soni, Naresh Somani, Taruni Bhattacharya, Nilesh Kucha

**Affiliations:** 1 Radiation Oncology, Bhagwan Mahaveer Cancer Hospital and Research Centre, Jaipur, IND; 2 Radiation Oncology, All India Institute of Medical Sciences, Jodhpur, IND; 3 Medical Oncology, Bhagwan Mahaveer Cancer Hospital and Research Centre, Jaipur, IND

**Keywords:** carcinoma prostate, mesothelioma, second malignancy

## Abstract

Pleural mesothelioma and carcinoma prostate as metachronous double malignancy is extremely rare co-occurrence. A 67-year-old male, diagnosed case of carcinoma prostate with bone metastasis, was treated with chemotherapy and hormone therapy. He responded well to chemotherapy and hormone therapy. He remained asymptomatic for two years with serum prostate-specific antigen (PSA) values within normal limits. After two years of diagnosis of carcinoma prostate, he developed left lung pleural sarcomatoid mesothelioma as a second metachronous tumor. Malignant pleural mesothelioma as a metachronous second tumor in a case of carcinoma prostate is rarely reported in the literature. The long-life expectancy, old age, late effects of the treatment, genetic predisposition and lifestyle factors of patients with carcinoma prostate expose them to the possibility of developing second primary tumor.

## Introduction

With the improvements in chemotherapy, hormone therapy and techniques of radiotherapy, the 10-year relative survival of patients diagnosed with prostate cancer has increased up to 98% [1]. The long-life expectancy, old age, late effects of the treatment, genetic predisposition and lifestyle factors of these patients expose them to the possibility of developing second primary tumor. In this case report, the extremely rare co-occurrence and association between prostate carcinoma and mesothelioma is discussed.

## Case presentation

**Figure 1:**
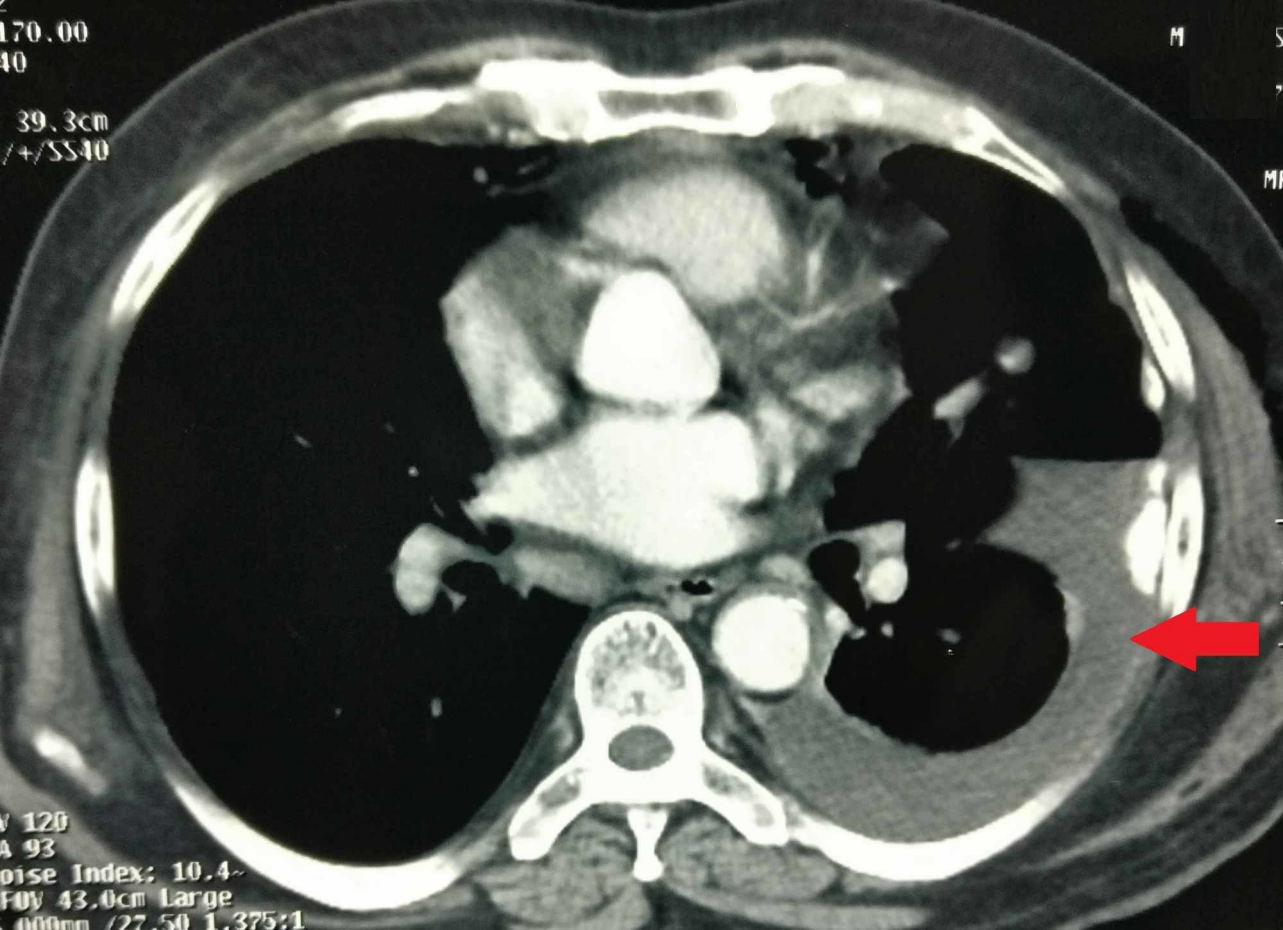
Contrast enhanced CT scan of chest showing left side pleural effusion, hydropneumothorax with resultant partial collapse of left lung, left lung pleural thickening with calcification and a pleural-based nodule measuring 17 x 13 mm in left upper lung.

A 67-year-old male, retired as security officer of copper mines area, presented with complaints of difficulty in micturition and backache since last four months. MRI pelvis showed enlarged prostate, altered signal intensity in peripheral zone of prostate gland predominantly on right side, involving adjacent part of right seminal vesicle, with irregularity of the prostatic capsule and right side iliac bone sclerotic metastatic lesion. Serum prostate-specific antigen (PSA) value was 67.69 ng/ml. Core needle biopsy from the prostate lesion was as moderately differentiated adenocarcinoma, Gleason’s score 3 + 4 = 7, perineural invasion was present, tumor was present in all cores. Bone scan showed right iliac bone metastasis lesion. Contrast enhanced CT scan chest and ultrasonography of abdomen were normal with no evidence of any metastatic lesion. He was diagnosed as carcinoma prostate with right iliac bone metastasis. He was treated with six cycles of docetaxel chemotherapy and denosumab, bicalutamide and gonadotropin releasing hormone (GnRH) agonist goserelin. Serum PSA levels came to 0.016 ng/ml after three months of initiation of the therapy. The hormone therapy was continued for the next two years and he remained asymptomatic for this period. After two years of the diagnosis of the prostate cancer he presented with difficulty in breathing, pain in left chest and backache for one week. Chest X-ray showed moderate left side pleural effusion. Left side inter-coastal drainage tube was inserted and pleural fluid aspiration was done. Pleural fluid cytology showed no malignant cells. Contrast enhanced CT scan of chest showed left side pleural effusion, hydropneumothorax with resultant partial collapse of left lung, left lung pleural thickening with calcification and a pleural-based nodule measuring 17 x 13 mm in left upper lung (Figure 1).

**Figure 2:**
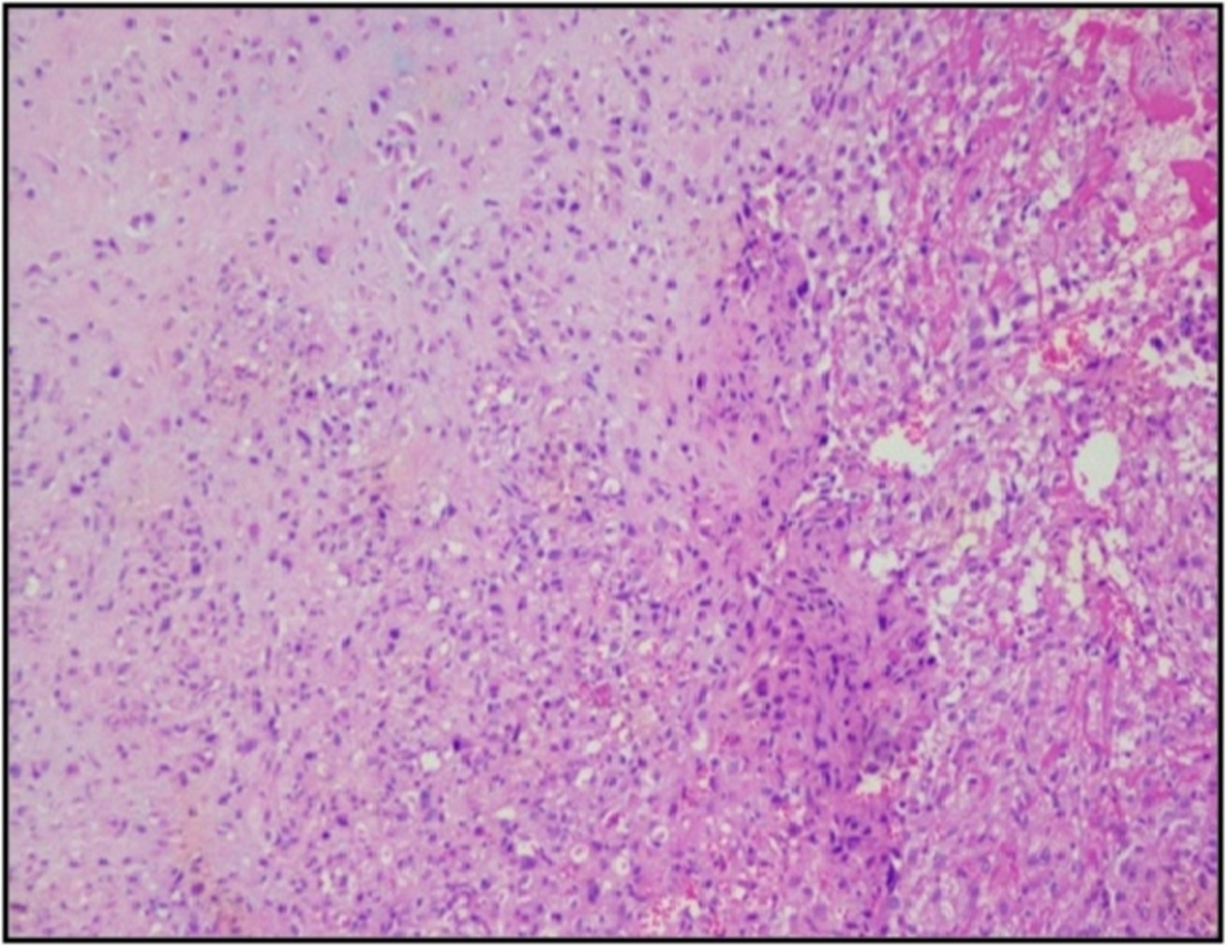
Microscopic characteristics (H&E stained, 10X view) of the left pleural nodule showing islands of cartilage surrounded by sheets of undifferentiated mesenchymal cells arranged in diffuse sheets with pleomorphic hyperchromatic nuclei with irregular nuclear borders and inconspicuous nucleoli and scant to moderate amount of cytoplasm. H&E: Hematoxylin & Eosin

Biopsy from the left pleural nodule by video-assisted thoracoscopic surgery (VATS) was done. The biopsy showed islands of cartilage surrounded by sheets of undifferentiated mesenchymal cells arranged in diffuse sheets with pleomorphic hyperchromatic nuclei with irregular nuclear borders and inconspicuous nucleoli and scant to moderate amount of cytoplasm (Figure 2).

**Figure 3:**
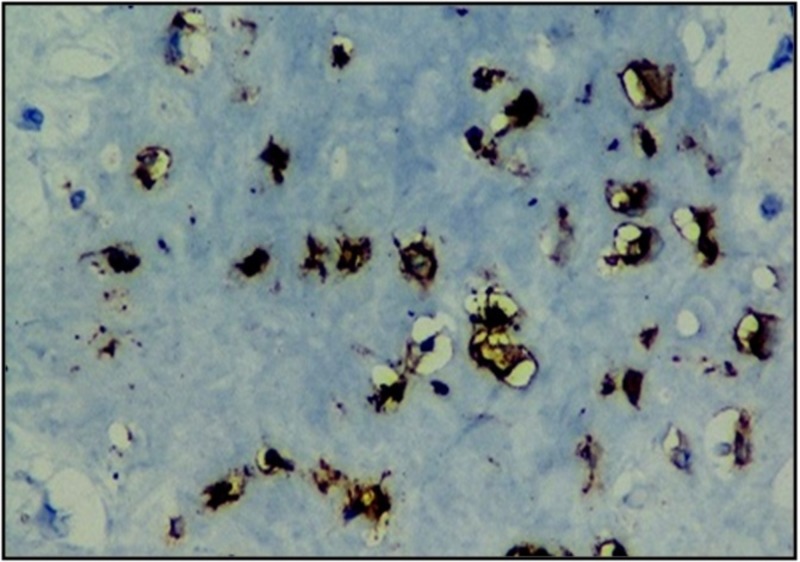
Immunohistochemistry analysis showing undifferentiated malignant mesenchymal tumor with positive staining for D2-40.

Immunohistochemistry staining was vimentin positive, S100 focally positive, WT-1 positive, CD 99 positive, D2-40 positive (Figure 3) while negative for Pan CK, S-100, NKX3.1, calretinin, PSA, P504, with Ki67 proliferation index of 80%, which was suggestive sarcomatoid mesothelioma with chondrosarcomatous differentiation.

**Figure 4:**
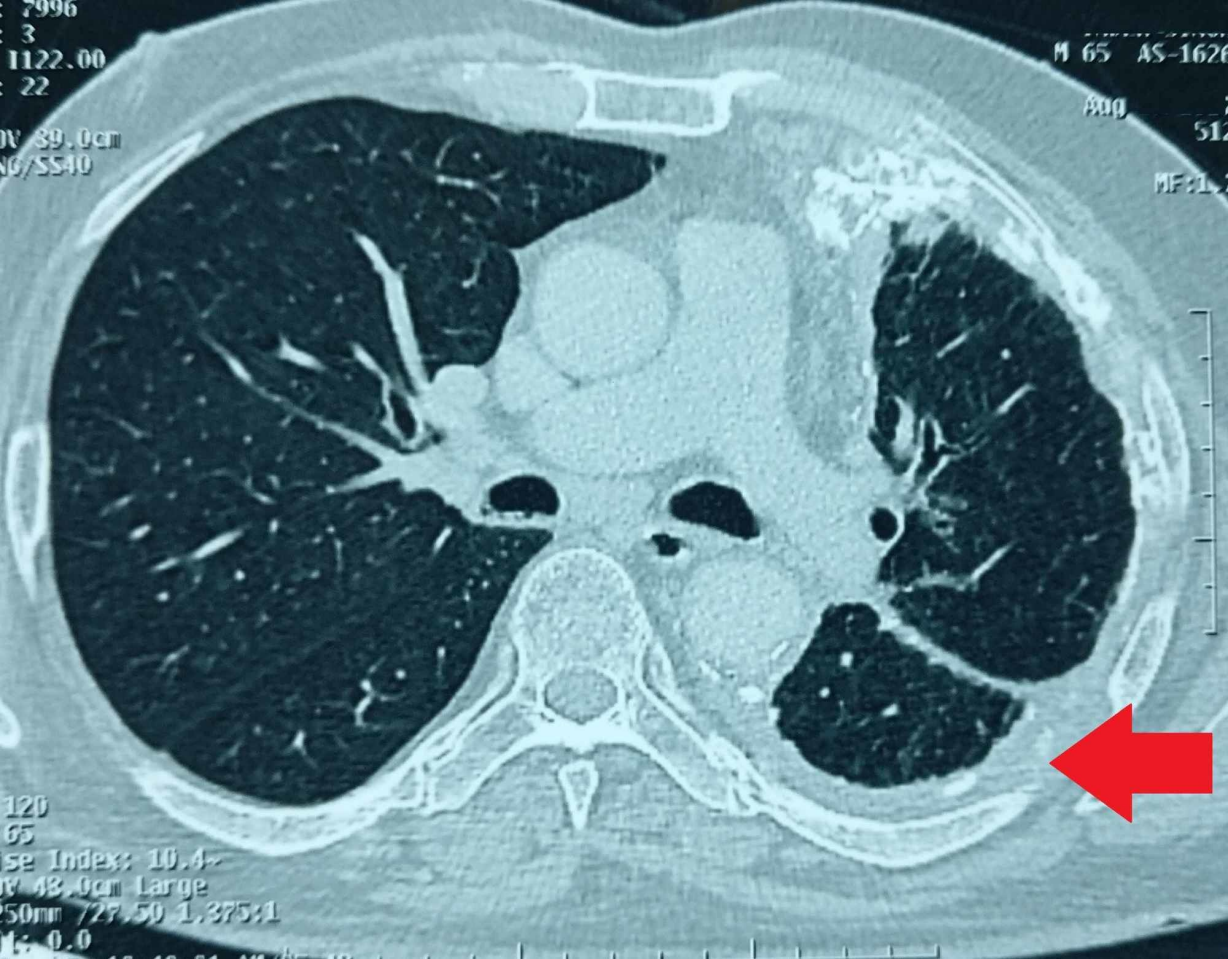
Contrast enhanced CT scan showing residual stable generalized nodular left lung pleural thickening with multifocal calcification, collapse and consolidation in left lung lower lobe, reticulo-nodular septal thickening and ground glass haze in left lung likely due to lymphangitic spread.

Poor prognosis was explained to the patient’s relatives, in view of aggressive mesothelioma as second malignancy. He was treated with six cycles of pemetrexed and carboplatin-based chemotherapy. PET-CT scan done for response assessment, showed FDG avid (SUV 7.44) residual stable generalized nodular left lung pleural thickening with multifocal calcification, collapse and consolidation in left lung lower lobe, reticulo-nodular septal thickening and ground glass haze in left lung likely due to lymphangitic spread (Figure 4).

He was advised for best supportive management with palliative care as further treatment.

## Discussion

The incidence of second primary malignancies (SPM) has increased. It comprises of the sixth most common cancer and makes 16% of all incident cancers burden [2]. SPM can be metachronous (second tumor diagnosed more than six months after the diagnosis of the primary tumor) or synchronous (second tumor diagnosed within six months of the primary tumor) [3]. The diagnostic criteria for SPM are that each tumor must present a definite picture of malignancy, each tumor must be distinct and the probability that one tumor is a metastatic lesion originating from the other must be excluded [4]. Cancer predisposition syndromes (Li Fraumeni and Beckwith-Wiedemann syndrome, Cowden syndrome and BRCA mutations, etc.), improved screening and diagnostic tests, increasing number of first cancer survivors, late adverse effects of chemotherapy and radiotherapy, life style, behavioral influences (tobacco, alcohol, etc.), old age and environmental exposures are among the factors leading to increase in incidence of SPM [5].

Effective and successful treatment with radiotherapy, surgery, chemotherapy and hormone therapy has increased the number of carcinoma prostate survivors. Prostate cancer patients have an increased risk of SPM [6-9]. In a Swedish National Prostate Cancer Register based study of 72,613 patients of prostate carcinoma, Van Hemelrijck et al. [6] found that about 17% of all prostate cancers associated in combination with another primary tumor. In this study, 6829 men diagnosed with another primary cancer before the diagnosis of prostate carcinoma, 138 men at the time of prostate carcinoma diagnosis, and 5230 men were diagnosed with another primary cancer after the diagnosis and treatment of prostate carcinoma. Cancers of the urinary bladder, colon and nonmelanoma of the skin were the three most frequently observed associated SPM [6].

In another Swiss study on population-based cancer registry, Rapiti et al. [7] found the overall standardised incidence rate (SIR) of SPM in prostate cancer patients treated with radiotherapy was 1.35 (p = 0.056). Braisch et al. [8], in a study of Bavaria (Germany) population-based cancer registry, evaluated 59,259 men diagnosed with carcinoma prostate between 2002 and 2008 and observed that the overall risk of SPM was increased significantly by 14% compared with the general population. Increased risk for second cancer of the bladder, kidney, pancreas, thyroid, small intestine and brain/nervous system was seen [8]. In a study of carcinoma prostate patients treated with radiotherapy, Moon et al. [9] found statistically significant increased odds of developing secondary cancers at several sites including bladder (odds ratio 1.63), rectum (odds ratio 1.63), colon, brain, stomach, melanoma, and lung cancer.

Mesothelioma is also associated with SPM probably via gene-asbestos exposure interaction. In a Swedish population-based registry, Chen et al. [10] found a bidirectional association of pleural mesothelioma with kidney cancer [for second kidney cancer after pleural mesothelioma: SIR = 4.4, 95% CI: 2.0-8.3; for second pleural mesothelioma after kidney cancer: SIR: 2.3, 95% CI: 1.3-3.9] according to the two-way analyses. He found significantly elevated risk for SPM [SIR = 1.4; 95% CI: 1.1-1.6] including ovarian cancer, small intestinal cancer, thyroid tumors and breast cancer, connective tissue cancer, and lung cancer associated with mesothelioma [10].

Many common chromosomal regions and genes are associated with prostate cancer and mesothelioma, which suggests the link between these two distinct tumors. Pathogenic variants and mutations in genes including BRCA1, BRCA2, HOXB13 and mismatch repair genes confer modest to high life time risk of prostate cancer [11, 12]. Prostate cancer in carriers of BRCA pathogenic variants is associated with aggressive disease such as higher Gleason score, higher tumor stage and/or grade at diagnosis [13]. Studies have suggested that genetic susceptibility including germline BAP1 mutations is associated with increased risk of mesothelioma probably through modulating asbestos exposure or via gene-asbestos exposure interaction [14, 15]. Tunesi et al. [14] conducted a gene-environment interaction analysis including asbestos exposure and 15 single nucleotide polymorphisms, and assessed gene-asbestos interaction on mesothelioma risk using relative excess risk due to interaction and synergy index for additive interaction and V index for multiplicative interaction. They found that gene-asbestos interaction has significant association with risk of mesothelioma susceptibility [14]. Recurrent somatic mutations in a number of tumor suppressor genes (i.e., cyclin-dependent kinase inhibitor 2A gene [CDKN2A], neurofibromin 2 (merlin) gene [NF2], and BRCA1 associated protein 1 gene [BAP1]) have been associated with mesothelioma [14, 15].

More than 80% of malignant mesothelioma patients have histories of asbestos exposure with incubation period between the onset of malignant mesothelioma and asbestos exposure about 30-40 years [16]. Asbestos is a unique mineral. It can be pulled apart into flexible fibers, and it is used for insulation, roofing, fireproofing, and sound absorption in many industries, for example, the building, cement, plastics and construction industries. Inhaled or ingested microscopic asbestos fibers, over the span of many years, cause genetic changes that can lead to asbestosis, lung cancer, mesothelioma and a number of other cancers like leukemia, esophageal, breast and prostate cancer [17]. Many studies have shown a potential link between asbestos exposure and prostate cancer [18-20]. Raffn et al. [18], in a Danish study on workers at an asbestos cement factory, found elevated number of prostate carcinoma cases with a 36% increase in the observed versus expected number of prostate cancers. Koskinen et al. [20], in a study of 23,285 male, asbestos production workers, found significantly elevated risk for lung cancer (SIR 1.14, 95% CI: 1.01-1.26), mesothelioma (SIR 2.77, 95% CI: 1.66-4.31), and prostate cancer (SIR 1.21, 95% CI: 1.09-1.34).

In summary, pleural mesothelioma and carcinoma prostate as double malignancy is extremely rare co-occurrence. The long-life expectancy, old age, late effects of the treatment, genetic predisposition and lifestyle factors of patients with carcinoma prostate expose them to the possibility of developing second primary tumor.

## Conclusions

Effective and successful treatment with radiotherapy, surgery, chemotherapy and hormone therapy has increased the number of carcinoma prostate survivors. Prostate cancer patients have an increased risk of second primary cancer. Mesothelioma as second malignancy in patients of carcinoma prostate is extremely rare. Further research is warranted to establish the association between carcinoma prostate and mesothelioma.
